# Editorial: Does healthcare financing explain different healthcare system performances and responses to COVID-19?

**DOI:** 10.3389/fpubh.2022.1062425

**Published:** 2022-11-03

**Authors:** Mihajlo Jakovljevic, Sulaiman Mouselli, Sanaa Al Ahdab, Dalal Hammoudi Halat

**Affiliations:** ^1^Institute of Advanced Manufacturing Technologies, Peter the Great St. Petersburg Polytechnic University, St. Petersburg, Russia; ^2^Institute of Comparative Economic Studies, Hosei University, Tokyo, Japan; ^3^Department of Global Health Economics and Policy, University of Kragujevac, Kragujevac, Serbia; ^4^Faculty of Business Administration, Arab International University Daraa, Daraa, Syria; ^5^Faculty of Pharmacy, AlBaath University Homs, Homs, Syria; ^6^School of Pharmacy, Lebanese International University, Khyara, Lebanon

**Keywords:** healthcare financing, healthcare system performance, COVID-19, out-of-pocket model, efficiency

The scale and impact of the COVID-19 pandemic are globally evident and present an unprecedented challenge to public health, causing devastating social and economic disruption as reported by the World Health Organization. There have been major differences in the responses and performance of national healthcare systems to the pandemic, and there is a need to thoroughly explore the roots of these variations.

The financing model is a distinguishing feature of any healthcare system. Despite the strong criticism of the out-of-pocket payment model that is common in Low-to-Middle Income Countries [LMICs; (1)], the performance of other models of healthcare systems in managing the pandemic is not necessarily superior (2). Surprisingly, recent attempts to examine the technical efficiency of healthcare systems suggest that a number of LMICs are outperforming those in developed economies (3).

The current pandemic represents a unique opportunity to explore whether the variation in the performance of healthcare systems to the pandemic is due to the healthcare financing model, or whether other factors have a greater influence on performance (4). Such an understanding is important in learning how to rethink the suitability of healthcare systems.

Yang et al. use a methodological framework, applying the two-agent game analysis. Their findings show that regional cooperation in facing the epidemic maximizes the regional benefits brought by containing the epidemic and improving economic performance. Yet, despite the differences in economic scale and epidemic prevention and control preferences between countries, not all of them directly benefitted from cooperation. Accordingly, the cooperative incentive of each country has to be strengthened through the allocation of cooperative income.

The second contribution entitled “The DEMATEL method explores the interdependent relationship structure and weights for diagnosis-related groups system” examines the overall performance of medical services. It indicates that patients and managers should first focus on the capacity of medical service providers when making a choice or deciding how to use the results of the DRGs system (Zou et al.).

Li et al. collected data from 3,098 A-share companies in 2019 and 2020. They applied the *Z*-score model to reasonably evaluate enterprise financial risk, and analyzed the impact of Research and Development (R&D) investment on enterprise financial risk under COVID-19. Their findings have revealed that the pandemic has increased the number of high-risk enterprises. R&D investment appears to be able to effectively reduce an enterprise's financial risk, and enterprises that attach importance to scientific research are relatively less affected by COVID-19. Compared with non-state-owned enterprises, R&D investment under state-owned enterprises can better help enterprises reduce financial risk. When the enterprise's financial risk is lower, the role of R&D investment in reducing financial risk is more significant.

An intriguing study came discusses Romania in eastern Europe, presenting a post-Semashko health system model. These authors tested working hypotheses using an econometric linear regression model based on the financing budgetary function, which matches funding to the specific need for each expenditure heading. The authors concluded that under the impact of pandemic stress, measures to improve healthcare management, increase performance and streamline financial allocation are vulnerable and cannot counteract the effects that the pandemic has on the healthcare of the population as reflected by the morbidity and mortality indicators collected during the pandemic (Antohi et al.).

Emerging BRICS (Brazil, Russia, India, China, and South Africa) countries, marked by long-term bold real GPD growth (5), are the strongest global drivers of supply and demand for medical goods, generic pharmaceuticals, and services outside wealthy OECD nations. The lockdowns triggered by the pandemic have strongly impacted airborne, naval, and land trade routes and ground supply chains among the major nations (6). The Global South's LMICs countries, many of which are represented in this Research Topic, present a huge diversity in terms of the historical legacy of their medical care financing and provision patterns (7). An array of clinical post-COVID-19 syndromes have worsened the burden of premature mortality and absenteeism attributable to chronic non-communicable diseases (8). The Global South's health systems face difficult sustainability issues, driven by these underlying patterns (9, 10). Their health system inefficiencies are becoming increasingly extended to the limits of resilience (5). Recent seminal evidence has documented that most of the supplies necessary for addressing COVID-19 and intensive care originate from China and other South-East Asian (ASEAN) nations (11).

The goal of this Research Topic was to explore the impact of financing on the readiness of different healthcare systems to respond to emergencies (12). Health policy considerations were primarily focused on financing mechanisms and healthcare system response (13). The editors believe that these heterogeneous contributions have opened new debates. The challenges faced during the COVID-19 pandemic, are here approached through the lens of several distinctively different world regions, including the Global South (9). We hope that this article Research Topic inspires scientific efforts in future epidemiology and public health developments.

## Author contributions

MJ prepared the manuscript draft. SM, SA, and DH revised the manuscript for important intellectual content. All authors fulfill ICMJE conditions for full authorship. All authors contributed to the article and approved the submitted version.

## Conflict of interest

The authors declare that the research was conducted in the absence of any commercial or financial relationships that could be construed as a potential conflict of interest.

## Publisher's note

All claims expressed in this article are solely those of the authors and do not necessarily represent those of their affiliated organizations, or those of the publisher, the editors and the reviewers. Any product that may be evaluated in this article, or claim that may be made by its manufacturer, is not guaranteed or endorsed by the publisher.

## References

[1] Jakovljevic M Potapchik E Popovich L Barik D Getzen TE. Evolving health expenditure landscape of the BRICS nations and projections to 2025. Health Econ. (2017) 26:844–52. 10.1002/hec.3406 27683202

[2] Jin H Li B Jakovljevic M. How China controls the Covid-19 epidemic through public health expenditure and policy? J Medical Econ. (2022) 25:437–49. 10.1080/13696998.2022.2054202 35289700

[3] Breitenbach MC Ngobeni V Aye GC. Global healthcare resource efficiency in the management of COVID-19 death and infection prevalence rates. Front Public Health. (2021) 9:638481. 10.3389/fpubh.2021.638481 33996718 PMC8116650

[4] Kumagai N. The impact of the COVID-19 pandemic on physician visits in Japan. Front Public Health. (2021) 9:743371. 10.3389/fpubh.2021.743371 34790642 PMC8591101

[5] Jakovljevic M Timofeyev Y Ranabhat C Fernandes PO Teixeira JP Rancic N. Real GDP growth rates and healthcare spending – comparison between the G7 and the EM7 countries. Glob Health. (2020) 16:64. 10.1186/s12992-020-00590-3 32677998 PMC7367257

[6] Grima S Rupeika-Apoga R Kizilkaya M Romānova I Dalli Gonzi R Jakovljevic M. A proactive approach to identify the exposure risk to COVID-19: validation of the pandemic risk exposure measurement (PREM) model using real-world data. Risk Manag Healthcare Policy. (2021) 14:4775. 10.2147/RMHP.S341500 34866947 PMC8637761

[7] Chattu VK Singh B Kaur J Jakovljevic M. COVID-19 vaccine, TRIPS, and global health diplomacy: India's role at the WTO platform. BioMed Res Int. (2021). 10.1155/2021/6658070 34485525 PMC8416375

[8] Jakovljevic M Jakab M Gerdtham U McDaid D Ogura S Varavikova E. Comparative financing analysis and political economy of noncommunicable diseases. J Medical Econ. (2019) 22:722–7. 10.1080/13696998.2019.1600523 30913928

[9] Jakovljevic M Liu Y Cerda A Simonyan M Correia T Mariita RM. The Global South political economy of health financing and spending landscape–history and presence. J Medical Econ. (2021) 24(Supp.1):25–33. 10.1080/13696998.2021.2007691 34866543

[10] Jakovljevic M Sugahara T Timofeyev Y Rancic N. Predictors of (in) efficiencies of healthcare expenditure among the leading Asian economies–comparison of OECD and non-OECD nations. Risk Manag Healthcare Policy. (2020) 13:2261–80. 10.2147/RMHP.S266386 33117004 PMC7585857

[11] Jakovljevic M Lamnisos D Westerman R Chattu VK Cerda A. Future health spending forecast in leading emerging BRICS markets in 2030: health policy implications. Health Res Policy Syst. (2022) 20:23. 10.1186/s12961-022-00822-5 35183217 PMC8857747

[12] Allahham L Mouselli S Jakovljevic M. The quality of Syrian healthcare services during COVID-19: a HEALTHQUAL approach. Front Public Health. (2022) 10:970922. 10.3389/fpubh.2022.970922 36033782 PMC9403656

[13] Al Ahdab S. A cross-sectional survey of knowledge, attitude and practice (KAP) towards COVID-19 pandemic among the Syrian residents. BMC Public Health. (2021) 21:1–7. 10.1186/s12889-021-10353-3 33546652 PMC7863039

